# Twenty cases of perennial and seasonal allergic rhinitis treated with LumiMed® Nasal Device

**DOI:** 10.1186/s13256-023-03980-4

**Published:** 2023-06-14

**Authors:** Denis Bouboulis, Avery Huff, Lauren Burawski

**Affiliations:** LumiMed, 106 Noroton Ave Suite 101, Darien, CT 06820 USA

**Keywords:** Allergic rhinitis, Phototherapy, Antihistamines, Corticosteroids, Photobiomodulation, Inflammation, Infrared

## Abstract

**Background:**

Allergic rhinitis is the most common allergic disease, with a prevalence up to 40% in the general population. Allergic rhinitis requires daily treatment to block inflammatory mediators and suppress the inflammatory response. However, these medications may have harmful side effects. Photobiomodulation as a treatment modality to reduce inflammation has been beneficial in many chronic disorders, yet therapy has not been US Food and Drug Administration approved for the treatment of allergic rhinitis. The LumiMed Nasal Device was designed to address the limitations associated with the treatment of allergic rhinitis with photobiomodulation. This in-office study hopes to show efficacy, usability, and comfortability of the LumiMed Nasal Device.

**Case presentation:**

Twenty patients with allergic rhinitis were treated during high allergy season with LumiMed Nasal Device. The average age of patients was 35 years (10–75); 11 were female and 9 were male. The population’s ethnicities were white (*n* = 11), Black (*n* = 6), Oriental (*n* = 2), and Iranian (*n* = 1). Patients were treated with twice-daily dosing, 10 seconds in each nostril, for 10 consecutive days. After 10 days, patients were evaluated for symptom relief, device comfort and device ease of use. The Total Nasal Symptom Score was used to assess severity of main symptoms of allergic rhinitis. The sum of Total Nasal Symptom Scores for each symptom category was calculated (total possible scores per patient were 0–9). Rhinorrhea/nasal secretions, nasal congestion, and nasal itching/sneezing were evaluated on a scale of 0–3 (0 no symptoms, 1 mild symptoms, 2 moderate symptoms, 3 severe symptoms). Device comfort was evaluated on a scale of 0–3 (0 no discomfort, 1 mild discomfort, 2 moderate discomfort, 3 severe discomfort). Device ease of use was evaluated on a scale of 0–3 (0 very easy, 1 somewhat difficult, 2 difficult, 3 very difficult).

**Conclusions:**

The results from these case studies indicated that of the 20 patients in this case study, 100% of patients experienced improvement in overall Total Nasal Symptom Score after using LumiMed Nasal Device. Of those patients, 40% brought their Total Nasal Symptom Score down to 0. Furthermore, 95% felt the LumiMed Nasal Device was comfortable to use, while 85% of patients felt the LumiMed Nasal Device was easy to use.

## Background

Allergic rhinitis (AR) is a common allergic disease with extraordinarily high prevalence in the general population and, consequently, a major economic burden in US healthcare costs. Specifically, AR is a disorder in which the immune system becomes sensitized to an aero-allergen and responds with a robust inflammatory response in the nasal mucosa upon the next exposure to that allergen. The prevalence of AR may be as high as 40% in the general population [1], and according to data from the National Health Interview Survey of 2018, provided by the National Center for Health Statistics at the Center for Disease Control and Prevention (CDC), the total number of adults and children diagnosed with AR in the preceding 12 months alone was nearly 30 million [2]. Rhinitis is also considered a major risk factor for the development of asthma, with up to 40% of people suffering from AR also having or having developed asthma [3, 4]. Moreover, the ability to control asthma in those also suffering from AR has demonstrated to be dependent upon controlling the rhinitis [5]. Given its prevalence in the general population, AR represents a consistent burden in US healthcare costs, with total costs of treating AR estimated at $11.2 billion [6]. Indirect costs associated with AR, particularly those associated with loss of productivity at work, have been estimated to be between $86 million to $7.7 billion [7]. Furthermore, the association between AR and asthma represents an even greater economic burden as it was estimated that the direct costs of asthma care are nearly $50 billion, with an additional $32 billion attributable to lost productivity from work and school absences [8].

### AR and the immune response

**Fig. 1:**
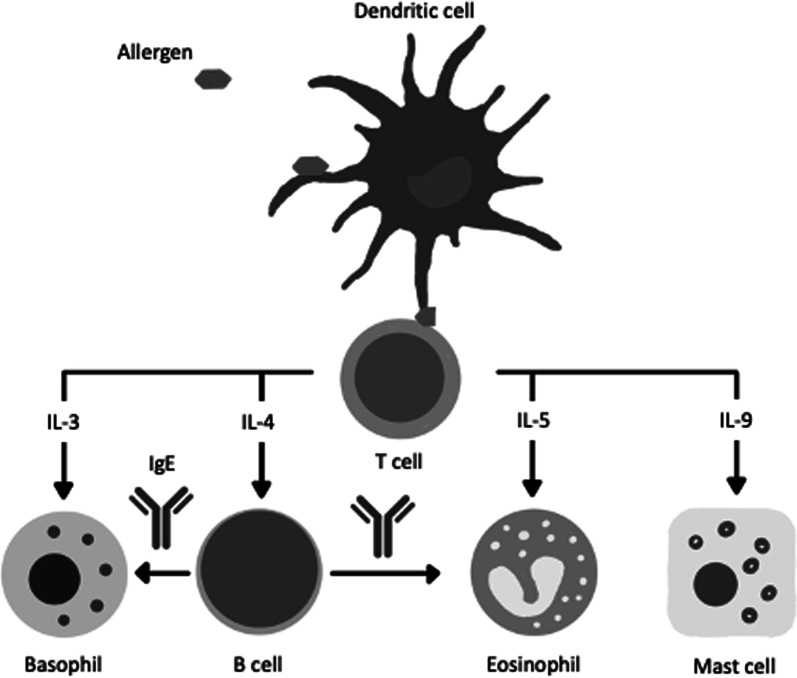
Allergen-induced sensitization. Allergens are processed by antigen presenting cells, such as dendritic cells in the mucosa, and allergen fragments are presented to naïve T cells. These newly activated Th2-type T cells secrete a number of cytokines that drive the proliferation of basophils, eosinophils, and mast cells, furthering allergic inflammation. B cells are also induced to produce allergen-specific IgE that binds to high affinity receptors on basophils and mast cells, facilitating the release of histamines, prostaglandins, and leukotrienes

AR is an allergen-stimulated and Immunoglobulin E-mediated inflammatory disease of the respiratory mucosa that can include both nasal and non-nasal symptoms. During initial exposure, an allergen captured by antigen-presenting cells in the respiratory tract is processed into peptides before being presented to naïve CD4^+^ T cells (Fig. 1). In individuals who are genetically predisposed to exhibit heightened immune responses to allergens, or atopic individuals, this peptide is recognized by CD4^+^ T cells. In particular, T helper (Th)2 cells capable of producing Th2-type cytokines, interleukin 4 (IL-4), interleukin 5 (IL-5), interleukin 9 (IL-9), and interleukin 13 (IL-13) are induced. Consequently, B cells recognizing the same peptides begin producing allergen-specific IgE antibodies. Mast cells, basophils, and eosinophils are the effector cells of allergic inflammation and possess high-affinity surface receptors for these IgE antibodies. The initial binding of IgE to these receptors is known as sensitization. Upon subsequent exposure to this particular allergen, mast cells and basophils would instantly degranulate, leading to the rapid release of pre-formed mediators including histamine, tryptase, chymase, and proteoglycans. Proteases, histamine, leukotrienes, and other cytokines are also produced following degranulation. Th2 cytokines, in particular, are essential mediators of allergic inflammation. IL-4 and IL-13 activate B cells, and IL-5 contributes to the activation of eosinophils. IL-13 also contributes to mucus production, generation of the extracellular matrix, and smooth muscle contraction [9–13]. The main nasal symptoms of AR are nasal pruritus, sneezing, rhinorrhea, and nasal congestion. Nasal pruritus and sneezing are induced by sensory nerve stimulation, whereas congestion results from vasodilation with resultant engorgement of cavernous sinusoids. Rhinorrhea can be induced by increased vascular permeability, as well as direct glandular secretion. Non-nasal symptoms that can be associated with AR include eye itching, eye tearing, itching of ears and/or palate, and eye redness [1]. The inflammation associated with AR is an IgE-dependent hypersensitivity to allergens.

### Current treatment strategies for AR

AR is commonly treated with daily pharmaceutical intervention to block the inflammatory mediators and suppress the inflammatory response driving symptomology. Such medications generally include antihistamines, leukotriene modifiers, decongestants, and corticosteroids. Many patients continue to suffer from AR annually, despite daily intervention with nasal corticosteroids and oral antihistamines, and seek relief options for their symptoms. Furthermore, patients who use oral antihistamines and nasal corticosteroids may not be fully aware of the side effects and long-term impacts associated with these medications. The commonly used intranasal corticosteroid has been implicated with anterior cataracts, headaches, nasal bleeding, and glaucoma. Furthermore, oral decongestants have been associated with tachycardia, increase in blood pressure, urinary retention, tremors, and insomnia. Antihistamines commonly cause sedation and cognitive impairment [14]. Finally, even when well tolerated, antihistamines and leukotriene modifiers are often efficacious in only 35% to 80% of patients [1].

Therefore, although numerous pharmacologic interventions exist for the treatment of AR, the risk of moderate to severe side effects and lack of efficacy in many patients creates an opportunity for innovative, non-pharmacologic treatment alternatives. These observations support the rationale of targeting the immune response to allergens with a novel treatment modality that will eliminate risks commonly associated with the standard of care for AR.

### Photobiomodulation: an innovative approach to the non-pharmacological treatment of AR

**Fig. 2:**
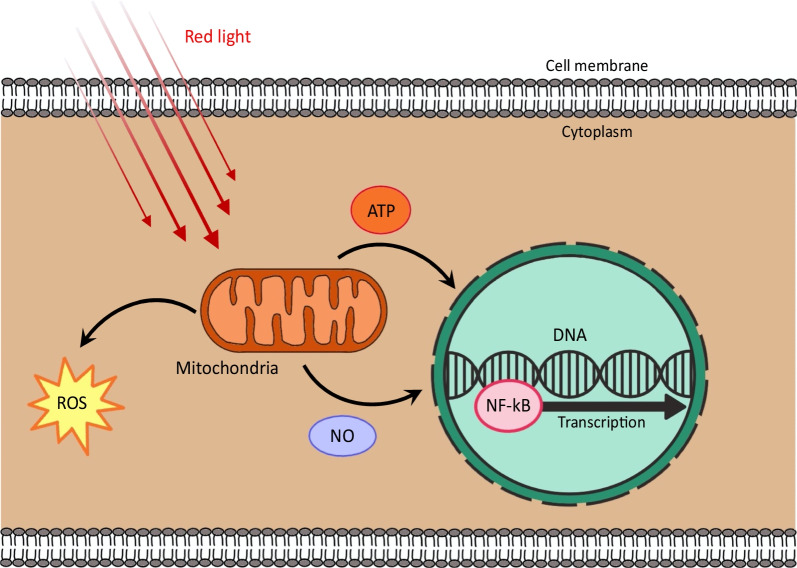
Mechanism of photobiomodulation. Red or near infrared light is absorbed by chromophores in the mitochondria. This triggers the production of ATP, reactive oxygen species, and nitric oxide. These factors collectively influence gene transcription through transcription factors like NF-kB, as well as other cellular processes, to decrease pain, alter proinflammatory cytokine profiles, regulate immune cells, etc. *ATP* adenosine triphosphate, *DNA* deoxyribonucleic acid, *NO* nitric oxide, *ROS* reactive oxygen species

Over the last 50 years, the incorporation of low-level laser therapy, known as photobiomodulation (PBM), has been shown to be beneficial in the reduction of inflammation across a wide range of disease states [15–18]. Specifically, the theory behind this treatment modality lies within its capacity to decrease inflammation in activated inflammatory cells through targeting cytochrome *c* oxidase in the mitochondria and light-sensitive opsins that modulate calcium ion channels (Fig. 2) [18]. This method serves to modulate several intermediary effects on reactive oxygen species, adenosine triphosphate, and calcium levels, which then activate transcription factors to elicit biphasic responses in cells that are dependent on both the dosimetry of the light and oxidative/inflammatory state of the cell [18]. At the appropriate dosage of 600 to 900 nm [19], PBM exhibits an extremely reproducible reduction in inflammation across tissue groups and disease states, including traumatic burn injury [20], arthritis [21–23], skeletal muscle (both injury and general functional improvement) [24, 25], traumatic brain [26, 27] and spinal cord [28] injury, and autoimmune diseases [29]. Of particular relevance to our proposed treatment of AR with intranasal phototherapy—and in support of such an approach—Silva and colleagues demonstrated that PBM reduced bronchial hyper-responsiveness, eosinophils, eotaxin, Th2 cytokines, and STAT6 levels in the lungs of mice with experimentally-induced asthma [30]. Moreover, several small studies in humans have also demonstrated the feasibility of intranasal phototherapy for the reduction of AR-associated symptoms [31, 32].

**Fig. 3:**
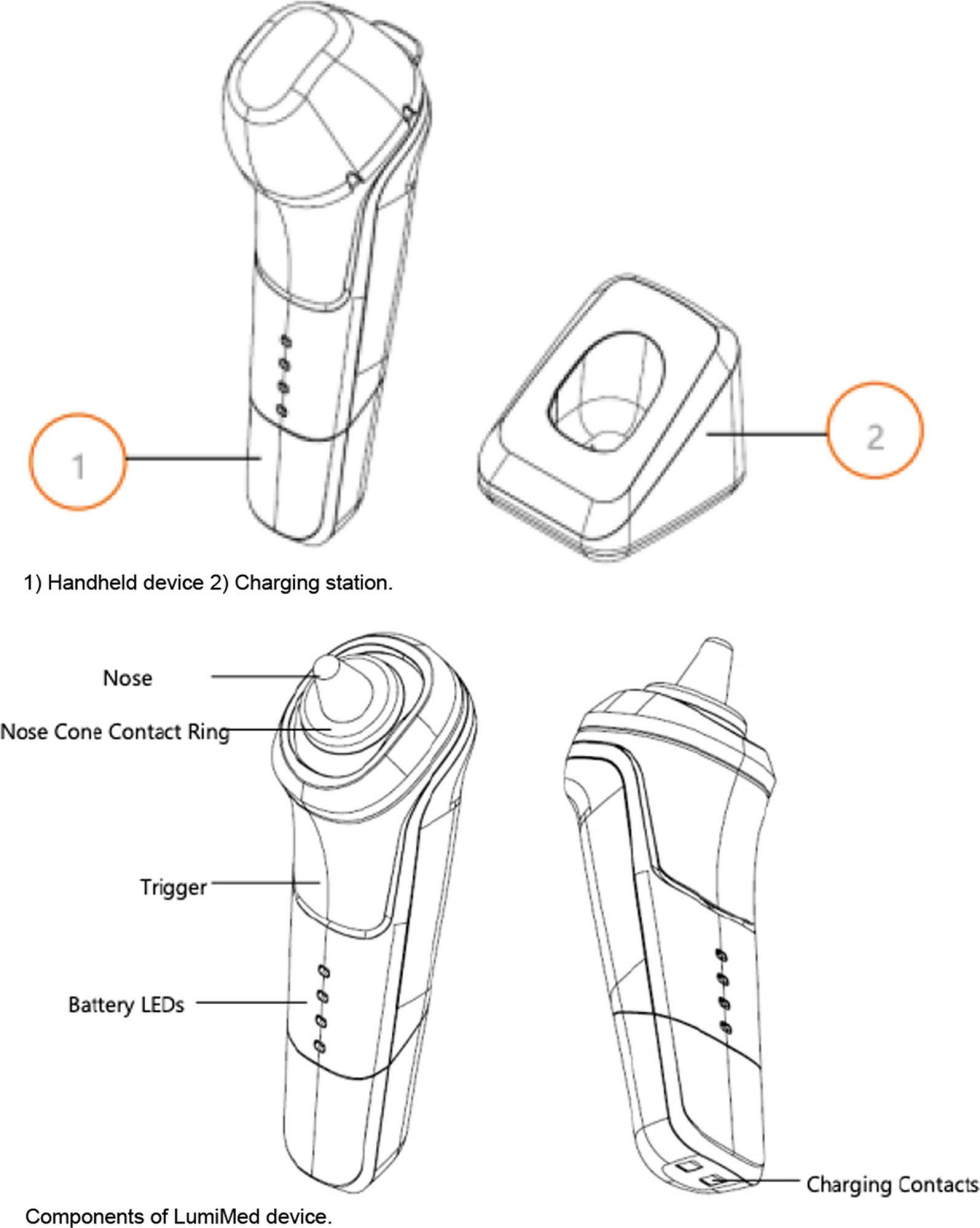
LumiMed phototherapy device

The LumiMed Nasal Device is a non-pharmacological, handheld, light-based allergy treatment device designed for adult and pediatric patients to treat seasonal and perennial AR by attenuating the allergen-induced inflammatory response (Fig. 3). With an intended daily use by patients, the primary use for the phototherapy device is to be used by patients with AR.

## Case presentation

Twenty patients with perennial and seasonal AR were treated during high allergy season with LumiMed Nasal Device. Patients were given instruction on how to use the LumiMed Nasal Device. Their average TNSS at baseline was 4.7 (Table 1).

**Table 1:** Patient scores at baseline

Patient number	Symptom category/score^a^			TNSS
	Rhinorrhea	Nasal congestion	Nasal itching/sneezing	
1	1	2	0	3
2	0	3	0	3
3	1	1	3	5
4	0	3	0	3
5	3	1	3	7
6	3	1	3	7
7	2	2	2	6
8	0	3	1	4
9	0	3	0	3
10	3	1	3	7
11	0	3	0	3
12	0	3	0	3
13	3	0	3	6
14	0	3	0	3
15	2	2	0	4
16	3	1	3	7
17	0	3	0	3
18	2	1	2	5
19	3	1	2	6
20	3	0	3	6

*TNSS* total nasal symptom score

^a^Rhinorrhea/nasal secretions, nasal congestion, and nasal itching/sneezing were evaluated on a scale of 0–3 (0 no symptoms, 1 mild symptoms, 2 moderate symptoms, 3 severe symptoms)

The light module in the LumiMed Nasal Device contains a light-emitting diode that creates an array of red light between 650 and 690 nm, which emerges from the device through an optically clear cone that is inserted into the patient’s nasal cavity by themselves. Each patient was expected to operate the LumiMed Nasal Device until 12 to 24 J/cm^2^ (energy required to generate 650 nm of light to stimulate PBM) of light energy has been emitted from the instrument (10 total seconds per nostril), twice per day for 10 days.

After 10 days, patients were evaluated for symptom relief, device comfort and device ease of use. Total Nasal Symptom Score (TNSS) was used for evaluation. The TNSS has been validated and adapted in most European countries, Canada, and the USA. It assessed the severity of main symptoms of the 20 patients with AR. The sum of scores for each symptom category was calculated (lowest score possible: 0 to highest score possible: 9). The sum of TNSS scores for each symptom category was calculated (total possible scores per patient were 0–9). Rhinorrhea/nasal secretions, nasal congestion, and nasal itching/sneezing were evaluated on a scale of 0–3 (0 no symptoms, 1 mild symptoms, 2 moderate symptoms, 3 severe symptoms). Device comfort was evaluated on a scale of 0–3 (0 no discomfort, 1 mild discomfort, 2 moderate discomfort, 3 severe discomfort). Device ease of use was evaluated on a scale of 0–3 (0 very easy, 1 somewhat difficult, 2 difficult, 3 very difficult).

### Patients

Patient 1 was a 15-year-old white female. She presented with symptoms of seasonal nasal congestion and runny nose in the spring. Her TNSS at baseline was 3 (rhinorhea: 1, nasal congestion: 2, nasal itching/sneezing: 0). After 10 days of treatment with LumiMed Nasal Device, her TNSS was 0 (rhinorhea: 0, nasal congestion: 0, nasal itching/sneezing: 0). Her device comfort score was 0. Her device ease of use score was 0.

Patient 2 was a 25-year-old white female. She presented with perennial nasal congestion predominantly in fall and winter “after the heat goes on in her house.” Her TNSS at baseline was 3 (rhinorhea: 0, nasal congestion: 3, nasal itching/sneezing: 0). After 10 days of treatment with LumiMed Nasal Device, her TNSS was 0 (rhinorhea: 0, nasal congestion: 0, nasal itching/sneezing: 0). Her device comfort score was 0. Her device ease of use score was 0.

Patient 3 was a 46-year-old white male. He presented with seasonal symptoms of sneezing, itchy nose, and mild nasal congestion. His TNSS at baseline was 4 (rhinorhea: 1, nasal congestion: 1, nasal itching/sneezing: 3). After 10 days of treatment with LumiMed Nasal Device, his TNSS was 1 (rhinorhea: 0, nasal congestion: 0, nasal itching/sneezing: 1). His device comfort score was 0. His device ease of use score was 0.

Patient 4 was a 75-year-old Iranian male. He presented with symptoms of perennial nasal congestion. His TNSS at baseline was 3 (rhinorhea: 0, nasal congestion: 3, nasal itching/sneezing: 0). After 10 days of treatment with LumiMed Nasal Device, his TNSS was 1 (rhinorhea: 0, nasal congestion: 1, nasal itching/sneezing: 0). His device comfort score was 1. His device ease of use score was 1.

Patient 5 was a 12-year-old white female. She presented with symptoms of itchy watery eyes and nose in early and late spring. Her TNSS at baseline was 4 (rhinorhea: 3, nasal congestion: 1, nasal itching/sneezing: 3). After 10 days of treatment with LumiMed Nasal Device, her TNSS was 1 (rhinorhea: 1, nasal congestion: 0, nasal itching/sneezing: 0). Her device comfort score was 0. Her device ease of use score was 0.

Patient 6 was a 32-year-old Black male. He presented with symptoms itchy eyes and nose and sneezing in the spring. His TNSS at baseline was 7 (rhinorhea: 3, nasal congestion: 1, nasal itching/sneezing: 3). After 10 days of treatment with LumiMed Nasal Device, his TNSS was 2 (rhinorhea: 0, nasal congestion: 1, nasal itching/sneezing: 1). His device comfort score was 0. His device ease of use score was 0.

Patient 7 was a 44-year-old white female. She presented with seasonal symptoms of nasal congestion, runny nose and itchy eyes. Her TNSS at baseline was 6 (rhinorhea: 2, nasal congestion: 2, nasal itching/sneezing: 2). After 10 days of treatment with LumiMed Nasal Device, her TNSS was 0 (rhinorhea: 0, nasal congestion: 0, nasal itching/sneezing: 0). Her device comfort score was 0. Her device ease of use score was 0.

Patient 8 was a 62-year-old white male. He presented with perennial symptoms of nasal congestion, which he said were “worse in the winter with his cat at home.” His TNSS at baseline was 4 (rhinorhea: 0, nasal congestion: 3, nasal itching/sneezing: 1). After 10 days of treatment with LumiMed Nasal Device, his TNSS was 1 (rhinorhea: 0, nasal congestion: 1, nasal itching/sneezing: 0). His device comfort score was 0. His device ease of use score was 0.

Patient 9 was a 41-year-old Black male. He presented with perennial symptoms of nasal congestion and postnasal drip. His TNSS at baseline was 3 (rhinorhea: 0, nasal congestion: 3, nasal itching/sneezing: 0). After 10 days of treatment with LumiMed Nasal Device, his TNSS was 0 (rhinorhea: 0; nasal congestion: 0; nasal itching/sneezing: 0). His device comfort score was 0. His device ease of use score was 0).

Patient 10 was a 22-year-old white female. She presented with seasonal symptoms of itchy, watery eyes, runny nose, which she said were “worse in spring and late fall.” Her TNSS at baseline was 7 (rhinorhea: 3, nasal congestion: 1, nasal itching/sneezing: 3). After 10 days of treatment with LumiMed Nasal Device, her TNSS was 2 (rhinorhea: 1, nasal congestion: 0, nasal itching/sneezing: 1). Her device comfort score was 0. Her device ease of use score 0.

Patient 11 was a 19-year-old Black female. She presented with seasonal symptoms of nasal congestion in late spring and early fall. Her TNSS at baseline was 3 (rhinorhea: 0, nasal congestion: 3, nasal itching/sneezing: 0). After 10 days of treatment with LumiMed Nasal Device, her TNSS was 0 (rhinorhea: 0, nasal congestion: 0, nasal itching/sneezing: 0). Her device comfort score was 0. Her device ease of use score was 0.

Patient 12 was a 10-year-old Oriental female. She presented with symptoms of chronic nasal congestion “with 2 cats at home.” Her TNSS at baseline was 3 (rhinorhea: 0, nasal congestion: 3, nasal itching/sneezing: 0). After 10 days of treatment with LumiMed Nasal Device, her TNSS was 1 (rhinorhea: 0, nasal congestion: 1, nasal itching/sneezing: 0). Her device comfort score was 0. Her device ease of use score was 0.

Patient 13 was a 25-year-old Black male. He presented with seasonal sneezing, watery nasal discharge in the spring. His TNSS at baseline was 6 (rhinorhea: 3, nasal congestion: 0, nasal itching/sneezing: 3). After 10 days of treatment with LumiMed Nasal Device, his TNSS was 2 (rhinorhea: 1, nasal congestion: 0, nasal itching/sneezing: 1). His device comfort score was 0. His device ease of use score was 0.

Patient 14 was an 11-year-old Oriental male. He presented with symptoms of perennial nasal congestion, “worse in the fall and winter.” His TNSS at baseline was 3 (rhinorhea: 0, nasal congestion: 3, nasal itching/sneezing: 0). After 10 days of treatment with LumiMed Nasal Device, his TNSS was 0 (rhinorhea: 0, nasal congestion: 0, nasal itching/sneezing: 0). His device comfort score was 0. His device ease of use score was 1.

Patient 15 was a 47-year-old white female. She presented with symptoms of nasal congestion and clear nasal discharge in early fall. Her TNSS at baseline was 4 (rhinorhea: 2, nasal congestion: 2, nasal itching/sneezing: 0). After 10 days of treatment with LumiMed Nasal Device, her TNSS was 1 (rhinorhea: 0, nasal congestion: 0, nasal itching/sneezing: 1). Her device comfort score was 0. Her device ease of use score was 0.

Patient 16 was a 59-year-old Black male. He presented with severe itchy watery eyes and nose with sneezing in spring. His TNSS at baseline was 7 (rhinorhea: 3, nasal congestion: 1, nasal itching/sneezing: 3). After 10 days of treatment with LumiMed Nasal Device, his TNSS was 1 (rhinorhea: 1, nasal congestion: 0, nasal itching/sneezing: 0). His device comfort score was 0. His device ease of use score was 0.

Patient 17 was a 65-year-old Black female. She presented with perennial nasal congestion that was “worse in the fall and winter.” Her TNSS at baseline was 3 (rhinorhea: 0, nasal congestion: 3, nasal itching/sneezing: 0). After 10 days of treatment with LumiMed Nasal Device, her TNSS was 0 (rhinorhea: 0, nasal congestion: 0, nasal itching/sneezing: 0). Her device comfort score was 0. Her device ease of use score was 1.

Patient 18 was a 37-year-old white female. She presented with itchy, watery eyes and nose in the spring and fall, but was “worse in the spring.” Her TNSS at baseline was 5 (rhinorhea: 2, nasal congestion: 1, nasal itching/sneezing: 2). After 10 days of treatment with LumiMed Nasal Device, her TNSS was 1 (rhinorhea: 0, nasal congestion: 0, nasal itching/sneezing: 1). Her device comfort score was 0. Her device ease of use score was 0.

Patient 19 was a 37-year-old white male. He presented with seasonal nasal congestion with sneezing, clear nasal discharge, and mild nasal congestion. His TNSS at baseline was 6 (rhinorhea: 3, nasal congestion: 1, nasal itching/sneezing: 2). After 10 days of treatment with LumiMed Nasal Device, his TNSS was 1 (rhinorhea: 1, nasal congestion: 0, nasal itching/sneezing: 0). His device comfort score was 0. His device ease of use score was 0.

Patient 20 was a 16-year-old white female. She presented with a watery, itchy nose in the spring. Her TNSS at baseline was 6 (rhinorhea: 3, nasal congestion: 0, nasal itching/sneezing: 3). After 10 days of treatment with LumiMed Nasal Device, her TNSS was 0 (rhinorhea: 0, nasal congestion: 0, nasal itching/sneezing: 0). Her device comfort score was 0. Her device ease of use score was 0.

The average baseline TNSS for the 20 patients in this case study was 4.7 and was reduced to 0.75 after 10 days of treatment with LumiMed Nasal Device (Tables 1 and 2). The results from these case studies indicated that of the 20 patients in this case study, 100% of patients experienced improvement in overall TNSS after using LumiMed Nasal Device. Of those patients, 40% brought their TNSS down to 0. Furthermore, 95% felt the LumiMed Nasal Device was comfortable to use, while 85% of patients felt the LumiMed Nasal Device was easy to use (Table 3).

**Table 2:** Patient scores 10 days post-treatment

Patient number	Symptom category/score^a^			TNSS
	Rhinorrhea	Nasal congestion	Nasal itching/sneezing	
1	0	0	0	0
2	0	0	0	0
3	0	0	1	1
4	0	1	0	1
5	1	0	0	1
6	0	1	1	2
7	0	0	0	0
8	0	1	0	1
9	0	0	0	0
10	1	0	1	2
11	0	0	0	0
12	0	1	0	1
13	1	0	1	2
14	0	0	0	0
15	0	0	1	1
16	1	0	0	1
17	0	0	0	0
18	0	0	1	1
19	1	0	0	1
20	0	0	0	0

*TNSS* total nasal symptom score

^a^Rhinorrhea/nasal secretions, nasal congestion, and nasal itching/sneezing were evaluated on a scale of 0–3 (0 no symptoms, 1 mild symptoms, 2 moderate symptoms, 3 severe symptoms)

**Table 3:** Patient-reported comfort and ease of use of LumiMed device

Patient number	Device comfort	Device ease of use
1	0	0
2	0	0
3	0	0
4	1	1
5	0	0
6	0	0
7	0	0
8	0	0
9	0	0
10	0	0
11	0	0
12	0	0
13	0	0
14	0	1
15	0	0
16	0	0
17	0	1
18	0	0
19	0	0
20	0	0

Device comfort was evaluated on a scale of 0–3 (0 no discomfort, 1 mild discomfort, 2 moderate discomfort, 3 severe discomfort). Device ease of use was evaluated on a scale of 0–3 (0 very easy, 1 somewhat difficult, 2 difficult, 3 very difficult)

## Discussion and conclusions

The LumiMed Nasal Device treatment modality is highly innovative over current forms of treatment for AR. Based on information from European studies, treatments from similar devices provide effective relief of symptoms of AR (equivalent to medication-based treatments). LumiMed Nasal Device provides a non-medication alternative for patients seeking relief from symptoms. Steroids and antihistamines have numerous side effects. There are no known side effects of this treatment device. LumiMed Nasal Device uses phototherapy to reduce allergen-induced histamines in the cells that line the nasal cavity, particularly mast cells, while also reducing inflammation of the mucus membranes. LumiMed Nasal Device is designed to deliver rapid treatments (in seconds) in each nostril, one to two times per day.

The results from these case studies indicated that of the 20 patients in this case study, 100% of patients experienced improvement in overall TNSS after using LumiMed Nasal Device. Of those patients, 95% felt the LumiMed Nasal Device was comfortable to use, while 85% of patients felt the LumiMed Nasal Device was easy to use. This case study shows that LumiMed Nasal Device is an effective, easy-to-use device for AR.

## Data Availability

Data sharing is not applicable to this article as no datasets were generated or analyzed during the current study.

## Abbreviations

AR: Allergic rhinitis
TNSS: Total nasal symptom score
PBM: Photobiomodulation
